# Effect of Amorphous Crosslinker on Phase Behavior and Electro-Optic Response of Polymer-Stabilized Blue Phase Liquid Crystals

**DOI:** 10.3390/nano12010048

**Published:** 2021-12-24

**Authors:** Kyung Min Lee, Urice Tohgha, Timothy J. Bunning, Michael E. McConney, Nicholas P. Godman

**Affiliations:** 1 Materials and Manufacturing Directorate, Air Force Research Laboratory, Wright-Patterson AFB, OH 45433, USA; urice.tohgha.ctr@us.af.mil (U.T.); timothy.bunning@us.af.mil (T.J.B.); Michael.McConney.1@us.af.mil (M.E.M.); 2 Azimuth Corporation, Fairborn, OH 45431, USA

**Keywords:** blue phase liquid crystals, polymer stabilization, amorphous crosslinker, electro-optic responses

## Abstract

Blue phase liquid crystals (BPLCs) composed of double-twisted cholesteric helices are promising materials for use in next-generation displays, optical components, and photonics applications. However, BPLCs are only observed in a narrow temperature range of 0.5–3 °C and must be stabilized with a polymer network. Here, we report on controlling the phase behavior of BPLCs by varying the concentration of an amorphous crosslinker (pentaerythritol triacrylate (PETA)). LC mixtures without amorphous crosslinker display narrow phase transition temperatures from isotropic to the blue phase-II (BP-II), blue phase-I (BP-I), and cholesteric phases, but the addition of PETA stabilizes the BP-I phase. A PETA content above 3 wt% prevents the formation of the simple cubic BP-II phase and induces a direct transition from the isotropic to the BP-I phase. PETA widens the temperature window of BP-I from ~6.8 °C for BPLC without PETA to ~15 °C for BPLC with 4 wt% PETA. The BPLCs with 3 and 4 wt% PETA are stabilized using polymer networks via in situ photopolymerization. Polymer-stabilized BPLC with 3 wt% PETA showed switching between reflective to transparent states with response times of 400–500 μs when an AC field was applied, whereas the application of a DC field induced a large color change from green to red.

## 1. Introduction

Blue phase liquid crystals (BPLCs), a three-dimensional photonic crystal structure, are highly twisted chiral nematic (or cholesteric) liquid crystals. The director axes self-assemble into double-twisted cylinders (DTCs) with a diameter of several 100 nm [1,2,3,4,5,6,7,8,9,10,11,12,13,14,15,16]. In the blue phase, double-twisted cylinders and disclination defects coexist. The disclination defect is a discontinuous point where the cylinders are in contact [17]. During cooling from the isotropic phase, three blue phases can be observed as a function of the chirality of LC. Blue phase III (BP-III) is an amorphous DTC, whereas BP-II and BP-I phases are three-dimensional simple cubic (SC) and body-centered cubic (BCC) phases, respectively.

The periodic blue phases result in selective Bragg reflections in the visible wavelength range. It is well-known that periodic, self-assembled cholesteric liquid crystals (CLC) exhibit selective reflection with a pitch ($P_{0}$) expressed as $\lambda_{0}=\bar{n}\;\cdot \;P_{0}$ and $\Delta \lambda_{0}=\Delta n\;\cdot \;P_{0}$, where *λ*_0_ is the center of the wavelength, Δ*λ* is bandwidth, $\bar{n}$ is the average refractive index of the liquid crystal, $P_{0}$ is the cholesteric pitch length, and Δn is the birefringence of the liquid crystal [12,18]. Unlike the CLC phase, blue phases do not require an alignment layer and the reflection wavelengths depend on the crystal planes.

The selective reflection ($\lambda_{hkl}$) of the cubic blue phases can be expressed as [19]:

(1) $$\lambda =\frac{2\bar{n}a}{\sqrt{h^{2}+k^{2}+l^{2}}}$$

where $\bar{n}$ is the average refractive index, a is the lattice constant of blue phases, and h, k, and l are the Miller indices. The lattice constant a of BP-I with a BCC structure corresponds to one pitch length, and diffraction peaks appear at (110), (200), (211), etc. In BP-II with a SC structure, the lattice constant corresponds to half a pitch length, and diffraction peaks appear at (100), (110), etc. [19,20].

BPLCs have many attractive features, such as fast response (sub-millisecond), narrow reflection band, no need for alignment layer, and intrinsic wide viewing angle [20]. BPLCs with these properties are potentially used for fast electro-optical responses in switchable or tunable devices. There are also challenges to the use of BPLCs in these applications, such as limited temperature range (0.5–2 °C) due to the defect disclinations and multi-domain (or polycrystalline) structures. The multi-domain structure leads to poor optical properties, such as increased scattering loss.

BPLCs with a multi-domain polycrystalline structure composed of several small platelet domains have been widely studied to date [5,6,9]. The polycrystalline domains are formed by random nucleation (both homogeneous and heterogeneous nucleation) and continue to grow until they come to contact with each other, resulting in platelet textures. The crystal orientation is mismatched in adjacent domains, resulting in grain boundaries, and the randomly oriented multiple domains reduce optical properties, such as increased scattering loss. To improve optical properties, single color monocrystalline BPLC is preferred and can be achieved by several methods: a rubbed polyimide alignment surface [15], a photoalignment layer [21], a chemically patterned substrate [22,23], a controlled temperature gradient [24], and an electric field-induced aligned domain structure [11,25]. Monocrystalline BPLCs have a uniform crystallographic lattice orientation, but grain boundaries still exist. During domain nucleation and growth, the same BP lattice orientations are maintained. Compared to polycrystalline BPs, monocrystalline BPs have superior optical properties, such as high reflectivity, low hysteresis, and high electro-optic performance (lower driving voltage).

BPLCs have a thermodynamically unstable structure due to disclination defects, which causes a narrow temperature range for the blue phase and limits the optical applications of BPLCs. To widen the temperature range of BPs, several materials have been used, such as polymers, nanoparticles, and small molecules. These materials migrate to the disclination lines, stabilizing the various blue phases; polymer stabilization is the most widely investigated method [1,2,3,26]. In 1993, Kitzerow and colleagues demonstrated the first report of polymer-stabilized BPLCs, but no dynamic responses were reported [26]. Kikuchi et al. reported that polymer-stabilized BPLCs (PSBPLC) could widen the BP temperature range by more than 60 °C using a small amount of polymer (4–11 wt%) [2]. Monomers first concentrate within the defect lines, forming a stabilizing polymer network via in situ photopolymerization. These PSBPLCs show a sub-millisecond switching response upon the application of an electric field. In 2012, Castles et al. reported thermally stable PSBPLCs with a 25–50 wt% polymer network in the range of −125 °C to 125 °C using templated BPLCs [1]. The photonic applications of the templated BPLCs were also demonstrated, such as switchable mirrorless lasers and electro-optic devices.

Two types of electro-optic responses have been reported in BPLCs and PSBPLCs: (1) reflective to transparent switching [2,20]; and (2) red or blue tuning responses [3,20,27]. The application of the electric field aligns the liquid crystal molecules in BP cubic structures parallel to the applied electric field direction if the dielectric anisotropy of LC is positive (Δε > 0) and aligns them perpendicular to the electric field direction if Δε is negative (Δε < 0). The reorientation of the LCs induces birefringence. The induced birefringence in the low electric field region is described as the Kerr effect:

(2) $$\Delta n_{ind}=\lambda KE^{2}\;$$

where *λ* is the wavelength of incident light, *K* is the Kerr constant, and *E* is the applied electric field strength [28,29]. The induced birefringence increases linearly with the Kerr constant. The fast response (<1 ms) and induced birefringence at low electric field are quite attractive for display and photonics applications. However, the higher electric field leads to a lattice distortion, called the electrostriction effect, and shifts the reflection band to a longer or shorter wavelength according to Equation (1) [27,30]. At very high electric field strengths, BP becomes a chiral nematic and ultimately a nematic phase [31,32]. Recently, Manda et al. reported the large reversible red and blue tuning response of monodomain polymer-stabilized BPLCs [20]. The tuning response is explained as the deformation of the cubic lattice (electrostriction effect) as a function of the electric field. The monodomain samples with the BPLC lattice located along the (200) plane or the (211) plane show blue or red tuning response. The direction of the lattice distortion with respect to the applied electric field determines the shift of the reflection band to longer (red tuning) or shorter (blue tuning) wavelengths. The applied electric field distorts the (200) plane laterally, resulting in a blue shift of the reflection band, while the (211) plane is distorted in the electric field direction, resulting in a redshift of the reflection band.

In this study, we report a systematic study on the effect of an amorphous crosslinker (PETA) on the phase behavior of BPLCs and the electro-optic (EO) response of PSBPLCs. Various ratios of chiral mesogenic monomer to amorphous PETA crosslinker were used, and increasing amounts of PETA induces a direct transition from isotropic to BP-I phase upon cooling, without the formation of the BP-II phase. The amorphous crosslinker can form polymer networks in the disclination lines, whereas the chiral mesogenic monomer can form polymer networks within the BPLC cubic structures. The polymer-stabilized BP-I phase has significantly improved thermal stability and exhibits a reflective electro-optical response. The application of an AC field induces a reflective to transparent switching response with a sub-millisecond (400–500 μs) response time, while the application of a DC field induces a reversible large redshift of the reflection band from green to red. The dynamic optical responses enable polymer-stabilized BPLCs to be used as color filters and bandpass filters.

## 2. Materials and Methods

**Scheme 1:**
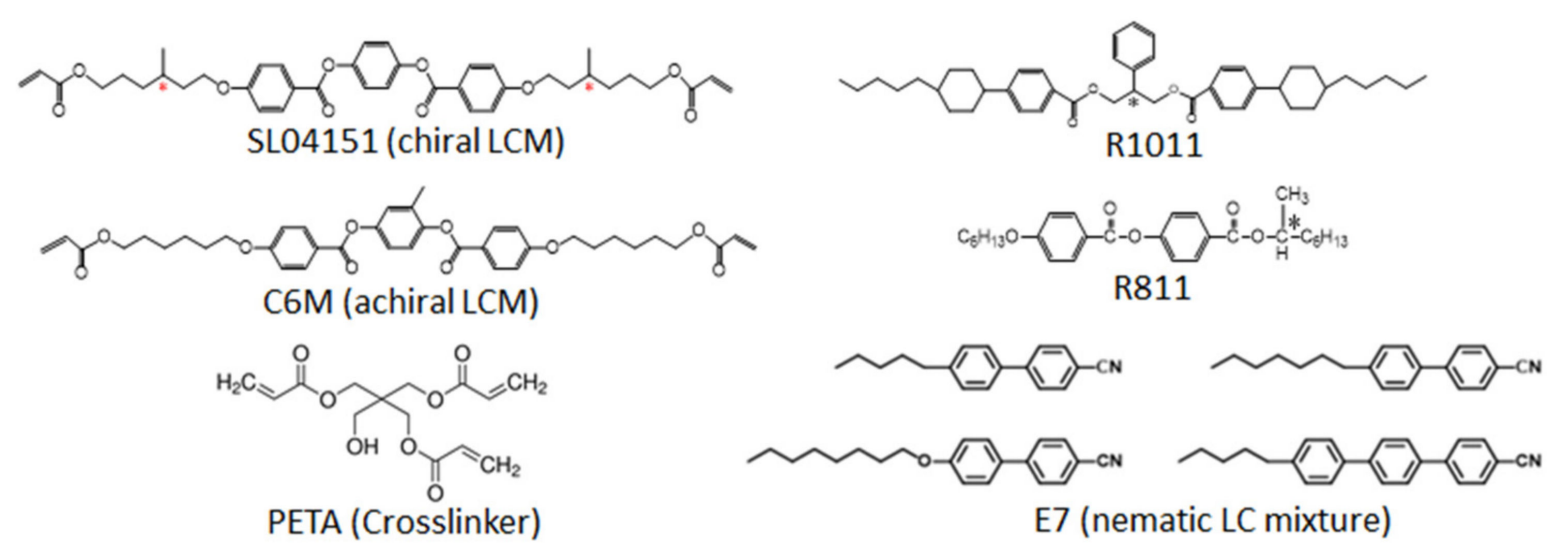
Chemical structures of the materials used in this study.

Materials: Five BPLC mixtures (BPLC-0, BPLC-1, BPLC-2, BPLC-3, and BPLC-4) were prepared using a chiral liquid crystal monomer (SL04151 from AlphaMicron Inc., Kent, OH, USA), pentaerythritol triacrylate (PETA from Sigma Aldrich, Milwaukee, WI, USA), chiral dopants (R1011 and R811 from Merck, Darmstadt, Germany), and a nematic mixture E7 (from Merck). The composition of these BPLC mixtures is summarized in Table 1 and the chemical structures are shown in Scheme 1. A total of 9 wt% of a monomer mixture was prepared by adjusting the ratio of chiral mesogenic monomer (SL04151) and amorphous crosslinker (PETA). The helical twisting power (HTP) of R1011, R811, and SL04151 with E7 is approximately 25, 8, and 12 μm^−1^, respectively.

Characterization: The phase behavior of the samples was monitored during cooling using an Ocean Optics spectrometer with an Instec temperature controller (mK1000, Naperville, IL, USA) and a polarized optical microscope (Nikon, Eclipse 50iPol, Tokyo, Japan) with a hot stage (Mettler FP-90, Columbus, OH, USA). The mixture was heated to an isotropic state and cooled slowly at a cooling rate of 0.1 °C min^−1^.

Cell preparation: Aligned or unaligned cells were prepared using indium tin oxide-coated glass slides (Colorado Concepts, Loveland, CO, USA). The glass substrates were cleaned with acetone and methanol and then treated with air plasma for 30 s. The substrates were spin-coated with 0.125 wt% Elvamide solution (Elvamide 8023R, DuPont, Wilmington, DE, USA). Elvamide solution was prepared by dissolving 0.125 g Elvamide in 0.975 g methanol at 40–45 °C overnight. The Elvamide alignment layer was rubbed with a cloth, and cells were constructed to ensure planar antiparallel alignment conditions (aligned cell). Unaligned cells were also constructed using Elvamide coated glass slides without rubbing. Cell gaps were controlled by mixing glass rod spacers (5–20 μm thickness) into an optical adhesive (NOA 65, Norland, Jamesburg, NJ, USA). The thickness of the cells was measured using an optical method based on the interference pattern of reflected light by the glass substrates in each empty cell.

Polymer-stabilized BPLCs: A small amount of photoinitiator (0.5 wt% Omnirad 651) was added to the BPLC mixture. The aligned or unaligned cells were filled with this mixture. The BP-I phase was stabilized by exposure to 365 nm wavelength UV light with an intensity of 1 mW cm^−2^ for 20 min.

## 3. Results and Discussion

### 3.1. Phase Behavior of BPLCs with Various PETA Concentrations

**Figure 1:**
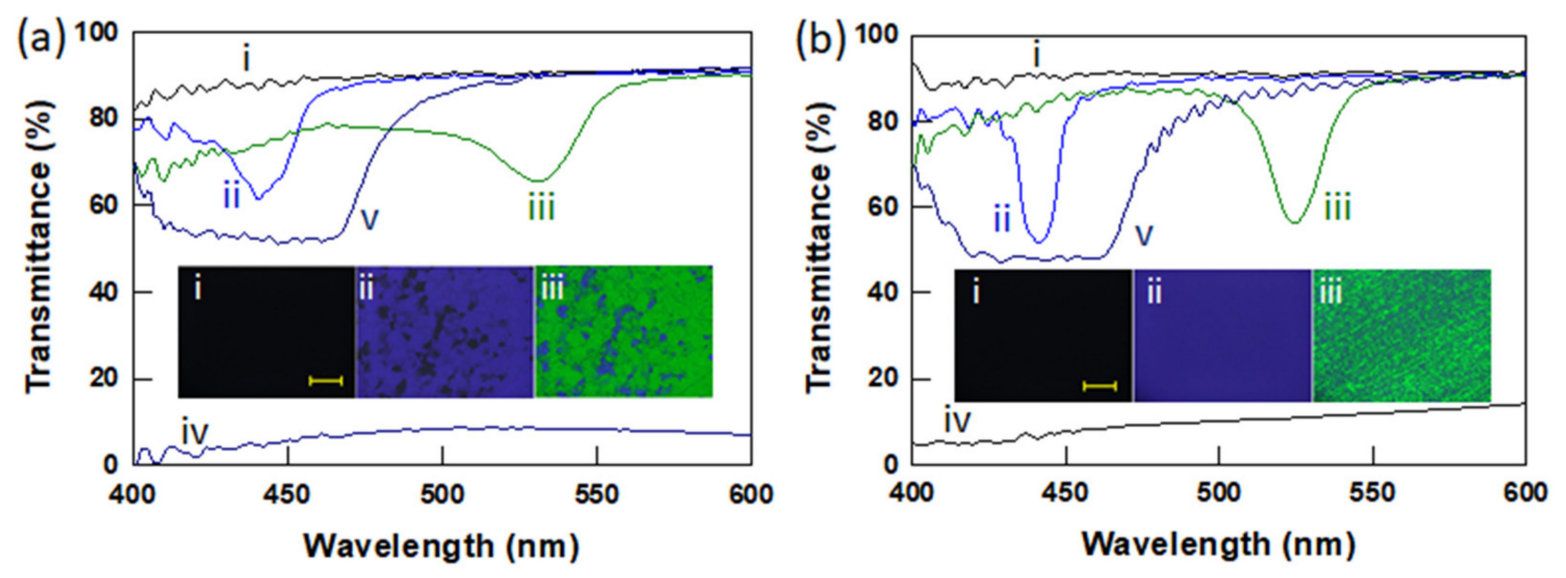
Transmission spectra and POM images of BPLC-0 prepared in (**a**) an unaligned cell at (**i**) 48.5 °C (isotropic), (**ii**) 47.3 °C (BP-II), (**iii**) 44 °C (BP-I), (**iv**) 35 °C (focal-conic phase), and (**v**) 24 °C (CLC phase after rubbing); and (**b**) an aligned planar cell at (**i**) 49 °C (isotropic), (**ii**) 47.8 °C (BP-II), (**iii**) 46.5 °C (BP-I), (**iv**) 35 °C (focal-conic phase), and (**v**) 24 °C (CLC phase after rubbing). Transmission spectra and POM images were monitored during cooling at a rate of 0.1 °C min^−1^. The LC mixture contains 9 wt% SL04151, 4 wt% R1011, 21 wt% R811, and 66 wt% E7. Scale bar in the inset POM images is 200 μm. Cells of 11.3 μm thickness were used.

The phase behavior of BPLC-0 containing 9 wt% SL04151, 4 wt% R1011, 21 wt% R811, and 66 wt% E7 in unaligned and aligned cells is shown in Figure 1. Four distinct phases are observed upon cooling at 0.1 °C min^−1^: isotropic, BP-II, BP-I, and cholesteric. The sample BPLC-0 prepared in an unaligned cell displays relatively broad BP-II and BP-I reflection peaks and multidomain platelet POM textures (Figure 1a). The multidomain texture and broad reflection bands are due to non-uniform nucleation, which results in domains with various crystal lattice orientations [3,19]. However, when BPLC-0 is prepared in the aligned planar cell, uniform nucleation is observed along the rubbing direction when cooled from the isotropic phase and produces BPLCs with uniform crystal orientation (monodomain structure) (Figure 1b) [13,16,21,33]. The initial nucleation state determines the crystal orientation of the BPs, and the addition of 1 wt% or 2 wt% amorphous crosslinking agent PETA (BPLC-1 and BPLC-2, respectively) resulted in identical phase transition behavior upon cooling as observed for BPLC-0 (Figure S1).

**Figure 2:**
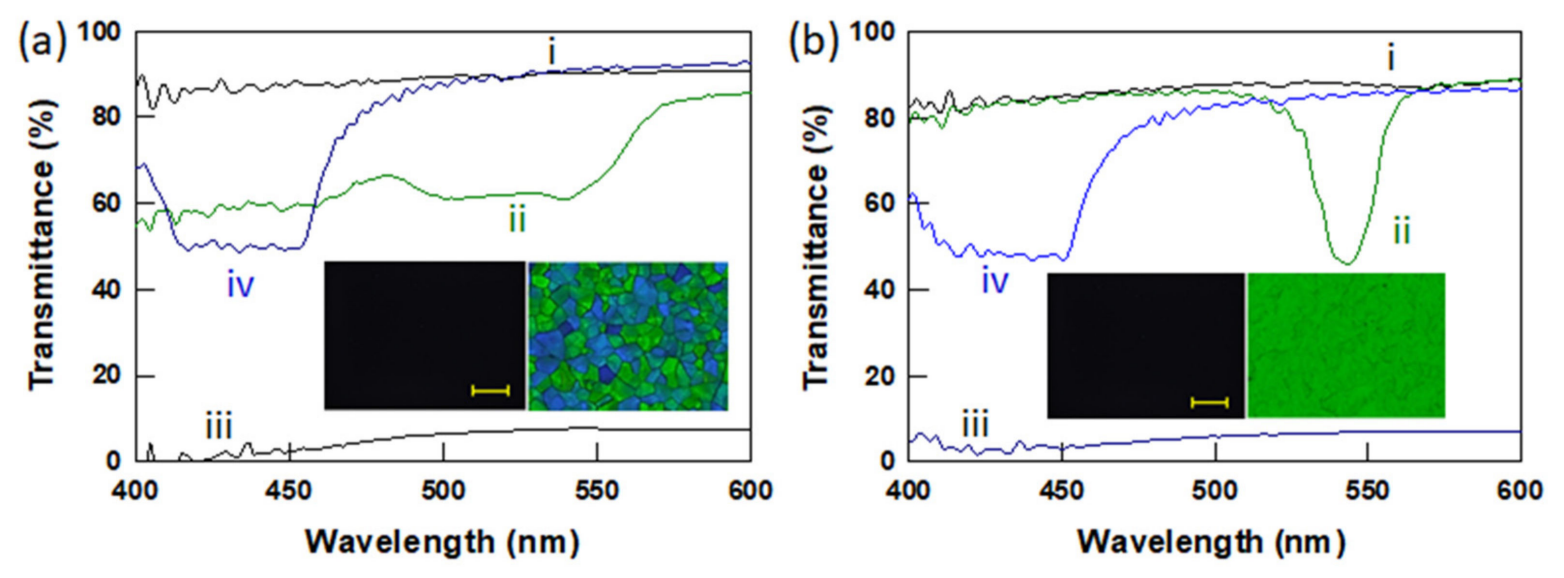
Transmission spectra and POM images of BPLC-3 prepared in (**a**) an unaligned cell at (**i**) 41 °C (isotropic), (**ii**) 26 °C (BP-I), (**iii**) 22 °C (focal-conic phase), and (**iv**) CLC phase after rubbing; and (**b**) an aligned cell at (**i**) 41 °C (isotropic), (**ii**) 26 °C (BP-I), (**iii**) 22 °C (focal-conic phase), and (**iv**) CLC phase after rubbing. Transmission spectra and POM images are monitored during cooling at a rate of 0.1 °C min^−1^. The LC mixture contains 6 wt% SL04151, 3 wt% PETA, 4 wt% R1011, 21 wt% R811, and 66 wt% E7. Scale bar in the POM images is 50 μm. Cells of 11.2 μm thickness were used.

Interestingly, a slight increase in amorphous crosslinker (3 wt% PETA, BPLC-3) resulted in a direct transition from the isotropic to BP-I phases without an intermediate transition through BP-II. This indicates that the ratio of the amorphous crosslinking agent (PETA) to the chiral mesogenic monomer (SL04151) has a great influence on the formation of the double twist cubic structures. Figure 2 shows the transmission spectra in unaligned and aligned cells, as well as POM images, wherein monodomain (uniform color and narrow bandwidth) and polydomain (platelets texture and broad bandwidth) BP-I textures of BPLC-3 are observed in aligned planar and unaligned cells, respectively. Compared to BPLCs with less than 2 wt% PETA, the BP-II phase of BPLC-3 is greatly suppressed and only the BP-I phase is observed upon cooling.

**Figure 3:**
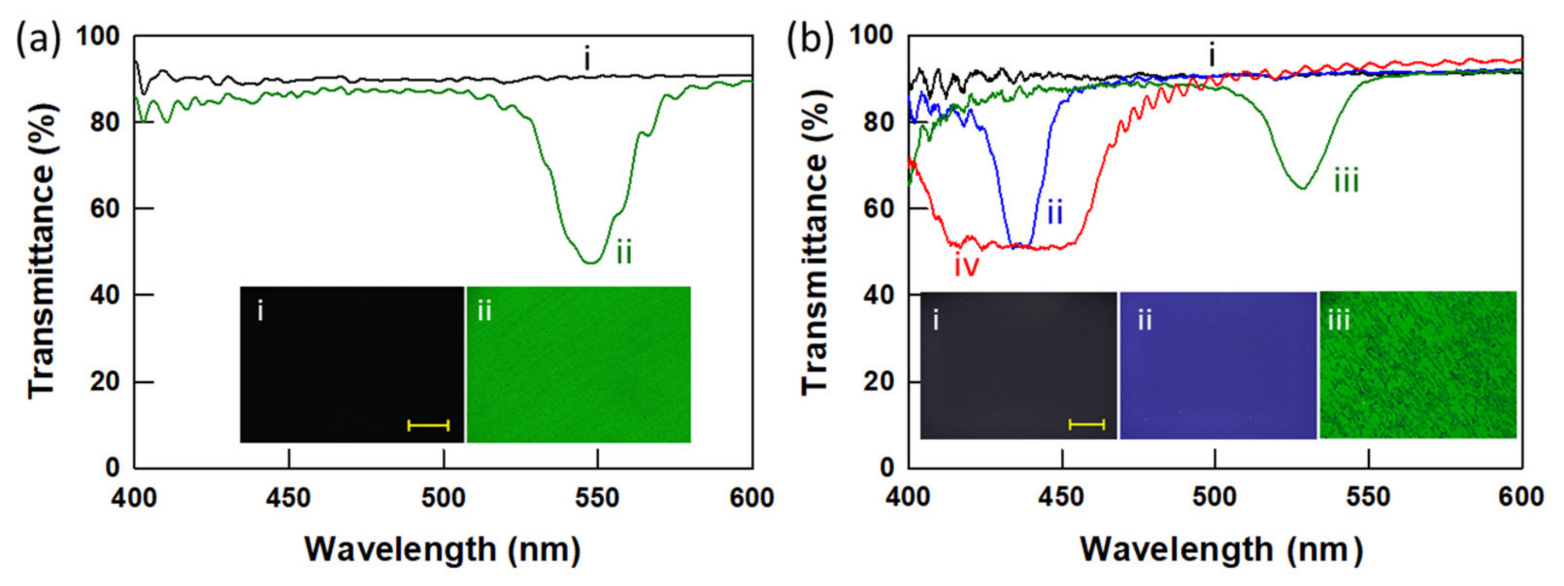
Transmission spectra and POM images of (**a**) BPLC-4 at (**i**) 36.5 °C (isotropic) and (**ii**) 25 °C (BP-I) and (**b**) BPLC-4-C6M at (**i**) 48 °C (isotropic), (**ii**) 46 °C (BP-II), (**iii**) 42 °C (BP-I), (**iv**) 24 °C (CLC). Both samples were prepared in an aligned planar cell. BPLC-4 contains 5 wt% SL04151, 4 wt% PETA, 4 wt% R1011, 21 wt% R811 and 66 wt% E7. BPLC-4-C6M contains 5 wt% SL04151, 4 wt% C6M, 4 wt% R1011, 21 wt% R811 and 66 wt% E7. Scale bar in the POM image is 200 μm. Cells of 11.3 μm thickness were used.

The phase behavior of BPLC-4 (4 wt% PETA) is similar to that observed for BPLC-3, direct isotropic to BP-I phase transition upon cooling, and the optical data and POM images are shown in Figure 3a. Interestingly, when an achiral mesogenic monomer (C6M) is used as a replacement for PETA (BPLC-4-C6M) both BP-II and BP-I phases are present upon cooling (isotropic—45.4 °C—BP-II—43.3 °C—BP-I), as depicted in Figure 3b. However, a more uniform green reflection with a narrower bandwidth and higher reflection efficiency are observed for BP-I samples prepared from the direct isotropic to BP-I transition (BPLC-4) compared to those of the BP-I samples prepared through the isotropic to BP-II and BP-I transition (BPLC-4-C6M). The stability of the BP-II simple cubic structure is greatly affected by the added amorphous crosslinking agent. The amorphous nonmesogenic monomer (PETA) is concentrated on the disclination lines due to immiscibility with LC (E7), while chiral or achiral mesogenic monomer dispersed in the LC medium [34,35]. An additional sample containing both achiral mesogenic C6M (5 wt%) and amorphous PETA (4 wt%) did not display BP-II behavior (isotropic—38.8 °C—BP-I). We tentatively conclude that the amorphous nonmesogenic crosslinker, which is concentrated mainly in the defect lines, greatly suppresses the formation of simple cubic BP-II structure and enhances the body-centered cubic BP-I structure.

**Figure 4:**
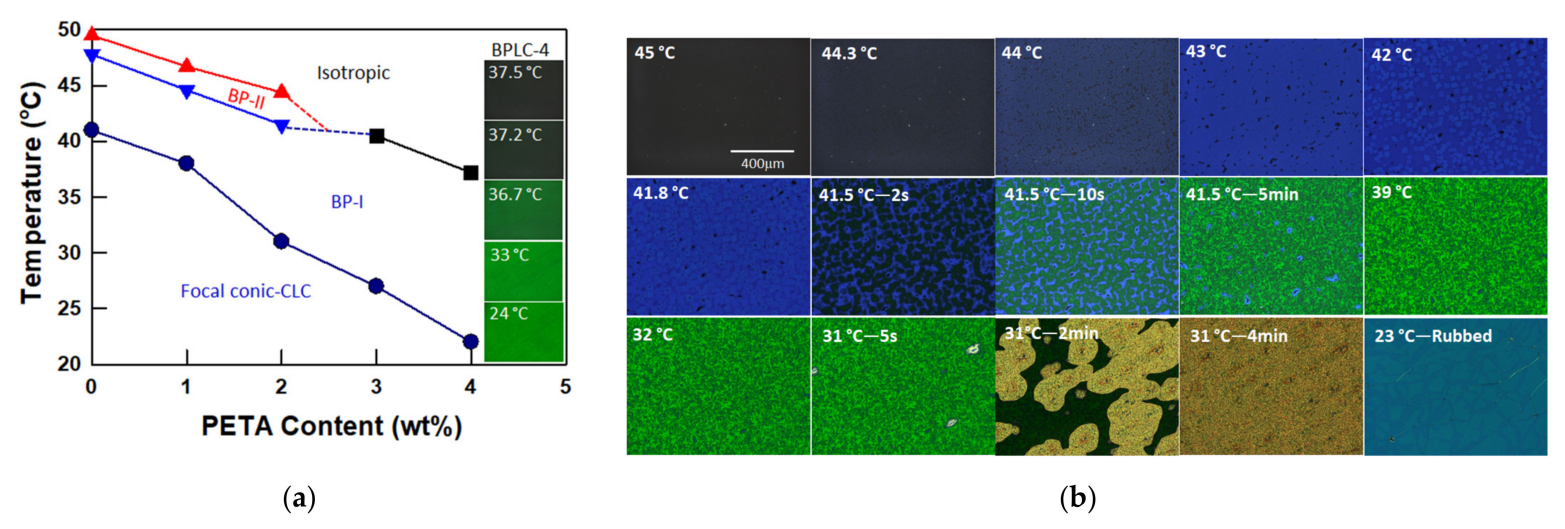
(**a**) Phase diagram: phase transition temperatures as a function of PETA concentration obtained from an Ocean Optics spectrometer and POM measurements. Insets are POM images of BPLC-4 on cooling. (**b**) POM images of BPLC-2 on cooling at a rate of 0.1 °C min^−1^.

A phase diagram of the BPLC mixtures is prepared using an Ocean Optics spectrometer and POM measurements (Figure 4a). The onset temperature was used as the phase transition temperature. The phase transition temperatures decrease with an increase in PETA concentration. It should be noted that the onset temperature of the cholesteric phase is difficult to determine due to the focal-conic phase that exists between the BPLC and the CLC phases. The focal-conic phase to CLC transition is very slow, as shown in Figure 4b. The BP-II phase of BPLC-2 appears at about 44.4 °C from the isotropic phase, the BP-I phase appears at about 41.5 °C, and the focal-conic phase is observed at about 31°C. The focal-conic phase is stable at room temperature (23–24 °C) for more than three weeks, and a planar CLC phase is only obtained after mechanical rubbing, which means that the cell with focal-conic phase is rubbed by hand. However, BPLC-4 shows two transitions, isotropic to BP-I transition at 37.2 °C and BP-I to focal-conic phase at around 22 °C. The POM images of BPLC-4 obtained during cooling are shown as insets in Figure 4a. A green color is observed from 36.7 °C to 24 °C and remains at room temperature (23–24 °C) for more than a week without polymer stabilization. The increased content of PETA widens the temperature window (ΔT) of the BP-I phase from ~6.8 °C for BPLC-0 to ~15 °C for BPLC-4.

As noted above, various monomers (PETA, chiral SL04151, and achiral C6M) are present within different portions of the blue phase cubic structures. The amorphous, nonmesogenic crosslinker migrates to the disclination lines to reduce the free energy penalty of the BP cubic structure, whereas the chiral mesogenic monomer is present in the double-twisted cylinders [20]. A small amount of PETA (0–2 wt%) does not affect the formation of the simple cubic BP-II structure. However, PETA concentrations greater than 3 wt% greatly destabilizes the BP-II structure and favor the BP-I. Small amounts of amorphous crosslinker of PETA (0–2 wt%) mainly dispersed in the disclination lines do not affect the formation of BP-II and BP-I phases. PETA concentrations greater than 3 wt% greatly inhibit the formation of BP-II and the formation of BP-I is energetically favored.

### 3.2. Polymer Stabilized BPLCs via In Situ Photopolymerization

**Figure 5:**
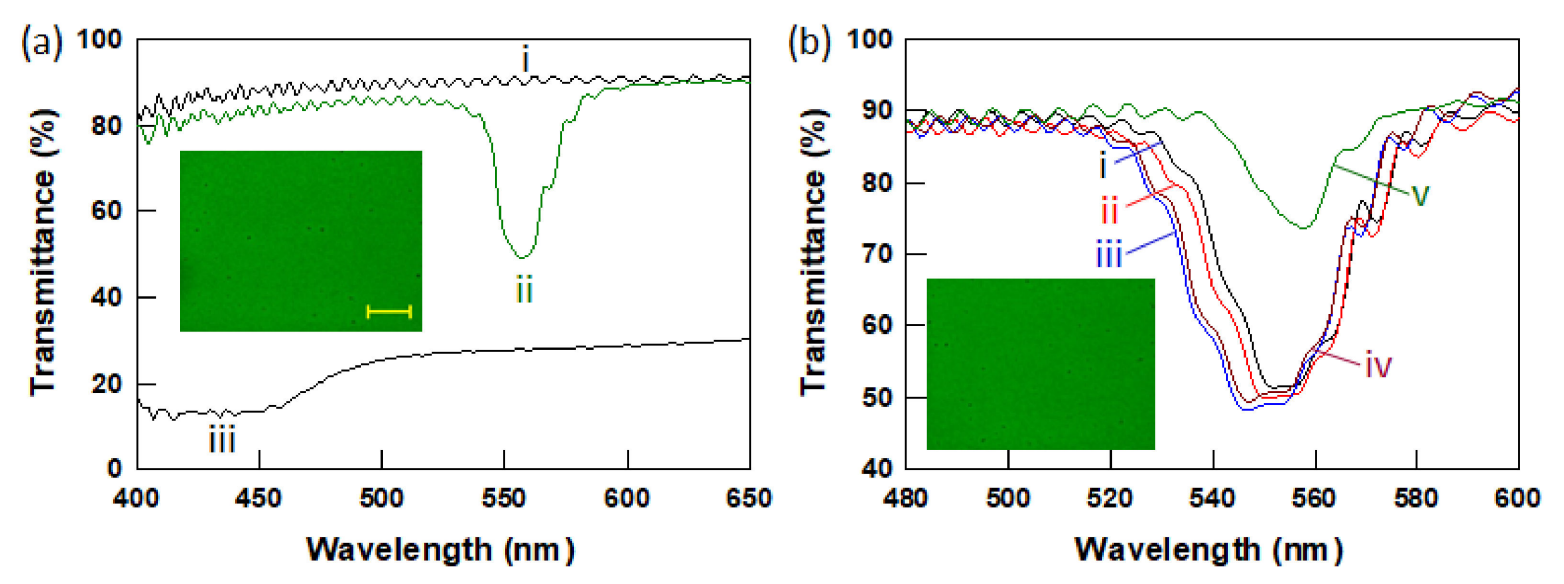
Polymer-stabilized BPLC-3 containing 0.5 wt% photoinitiator (Omnirad 651): (**a**) Before UV exposure at (**i**) 38.5 °C, (**ii**) 29 °C, and (**iii**) 20 °C; and (**b**) after UV exposure at 29 °C with 1 mW cm^−2^ 365 nm wavelength UV light for 20 min. Temperature decreases from (**i**) 29 °C, (**ii**) 25 °C to (**iii**) 3 °C, and increases to (**iv**) 10 °C, and (**v**) 40 °C. A cell with 11.3 μm thickness was used. Insets are POM images at 29 °C (**a**) before and (**b**) after UV curing. Scale bar in the POM image is 200 μm.

Two mixtures (BPLC-3 and -4) with excellent optical properties were selected to study the electro-optical response. A small amount of photoinitiator (Omnirad 651) was added to BPLC-3 and BPLC-4 mixtures to polymer-stabilize BP-I. Figure 5 shows the transmission spectra of BPLC-3 containing Omnirad 651 before and after photopolymerization. Two transitions are observed before UV curing: an isotropic to BP-I transition at 38.5 °C and a BP-I to focal-conic phase transition at 22 °C. The green reflective color observed at 29 °C is shown in the inset image of Figure 5a. This mixture is subsequently exposed to 365 nm wavelength UV light at 29 °C with an intensity of ~1 mW cm^−2^ for 20 min. The thermal stability of the polymer-stabilized BPLC-3 (PSBPLC-3) is greatly improved post polymerization (Figure 5b), and the BP-I phase of PSBPLC-3 is stable from 42 °C to at least 3 °C (ΔT > 39 °C). The BP-I phase of PSBPLC-3 might be stable at temperatures lower than 3 °C, but the cold water circulator employed could not cool to a lower temperature. While the low-temperature stability of BP-I is significantly improved, the high-temperature stability is only marginally improved. However, the high temperature stability is typically limited by the liquid crystal clearing temperature [2].

### 3.3. Electro-Optic Response of Polymer-Stabilized BPLCs

**Figure 6:**
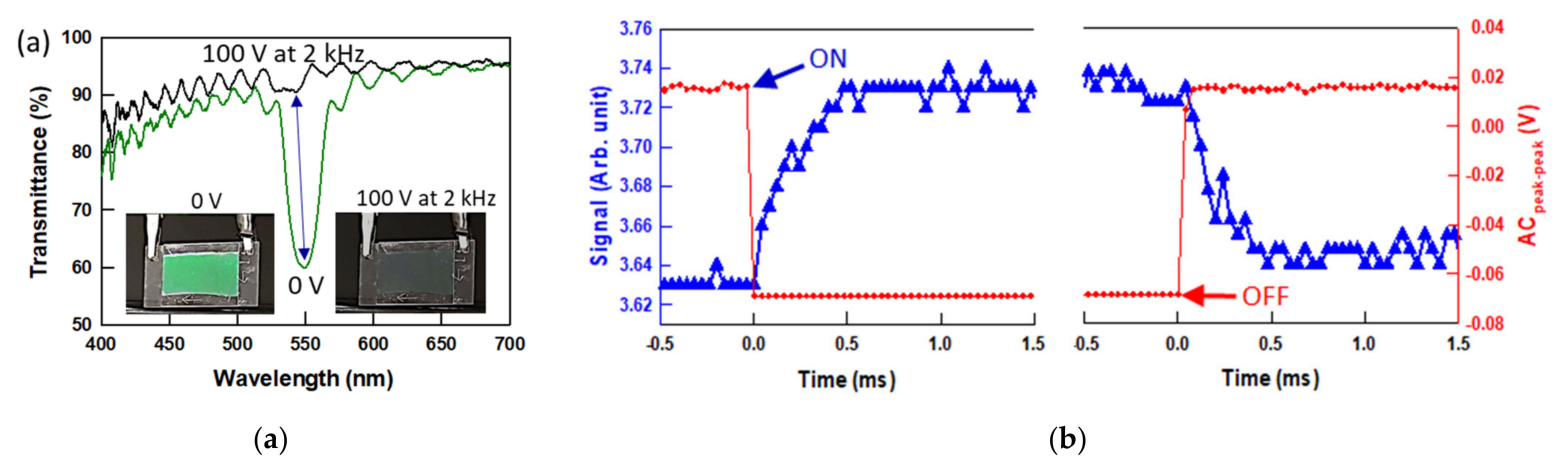
(**a**) Transmission spectra of the reflective to transparent switching response and green to transparent reflection color change (inset image) and (**b**) optical signal of 4.7 μm thick PSBPLC-3 when an AC voltage of 100 V at 2 kHz is turned on and off.

Application of an electric field induces a local reorientation of the liquid crystal medium in relatively low electric fields and electrostriction in high electric fields [28,29]. Figure 6 shows the reversible switching response between the reflective and transparent states of the PSBPLC-3 when an AC electric field of 100 V at 2 kHz is turned on and off. The decrease in transmittance when the AC voltage is increased by 10 V at 2 kHz is summarized in Figure S2. The green reflective color disappears when the AC field is directly applied to 100 V, and the color reappears when the voltage is removed (Figure 6a, insets) [2,34,35,36,37,38]. Figure S3 shows the transmittance of PSPLC-3 as the AC field increases stepwise (slowly) to 100 V at 2 kHz. This blue shift of the reflection band is caused by the rotation of the cubic structure and the transparent state is obtained from the reorientation of positive Δε E7 in BP-I. The switching times obtained by a 90/10% method are in the range of about 400–500 μs (rise and fall times). The rotation of LCs in BPs due to the short coherent length of BPLC leads to a fast response time (<1 ms) [2]. The amorphous polymer network in the declination line stabilizes the BPs and prevents the AC electric field-induced phase transition. Applying a high AC field induces a local reorientation and no electrostriction occurs.

**Figure 7:**
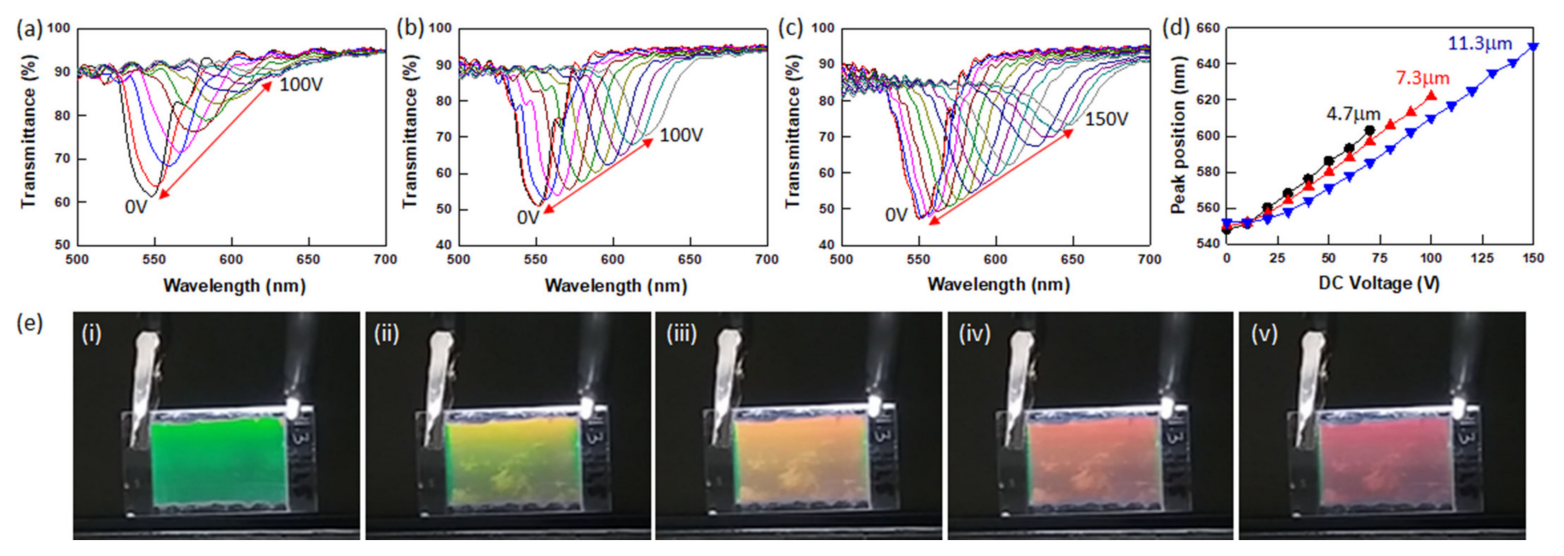
Red tuning response of PSBPLC-3 with (**a**) 4.7 μm, (**b**) 7.3 μm, and (**c**) 11.3 μm thicknesses with increasing DC voltage. (**d**) Summary of peak positions as a function of DC voltage. (**e**) Photographs of increasing DC voltages: (**i**) 0 V, (**ii**) 50 V, (**iii**) 90 V, (**iv**) 120 V, (**v**) 150 V DC.

Interestingly, when a direct current (DC) electric field is applied to PSBPLC-3 with various cell thicknesses, the reflection band shifts to longer wavelengths (Figure 7a–c) rather than switching to a transparent state. The tuning range of PSBPLC-3 as a function of cell thickness and DC voltage is summarized in Figure 7d. The DC voltage-induced color change of PSBPLC-3 (11.3 μm thick cell) from green to yellow to red as the DC voltage is increased to 150V is shown in Figure 7e. Application of a DC field induces lattice deformation (electrostriction), rather than a local reorientation, of the BP-I structure, and increasing the DC voltage shifts the photonic bandgap to longer wavelengths [7,20,27,39]. PSBPLC-3 with various cell thicknesses exhibits electrostriction coefficients ranging from 1.2–1.7 × 10^−3^ µm^2^ V^−2^ at low DC voltages and 3.6–8.4 × 10^−4^ µm^2^ V^−2^ at high DC voltages [40]. Additional information is shown in Supplementary Materials and Figure S4. The slope change indicates the lattice distortion of the BP-I phase and requires further study. As noted above, the nonmesogenic PETA stabilizes the disclination lines and improves thermal stability of BP, whereas the chiral mesogenic polymer (SL04151) is present throughout BP-I to stabilize the double-twisted cylinder structures. The positive Δε LCs in the body-centered cubic BP-I structure are strongly stabilized by SL04151. The nonmesogenic amorphous polymer concentrated in the disclination lines improves the thermodynamic stability of BP, but the amorphous polymer network does not form a chiral structure that can exhibit the electrostriction effect. Thus, achiral nonmesogenic polymers cause local reorientation (Kerr effect), resulting in a reflective to transparent switching response. Because the chiral polymer network forms a chiral structure, application of an AC [20,39,41] or DC [7,24,42] electric field induces lattice distortion and deformation in the BP structures, resulting in the shift of the photonic bandgap. There might be an optimal balance between chiral mesogenic and amorphous monomer concentrations to achieve both high stability and tunability [2,20]. As shown in Figure 6 and Figure 7, BPLC-3 shows both switching and red tuning responses due to the two different polymer networks. PSBPLC-4 prepared from a mixture containing 4 wt% PETA shows the same EO response as PSBPLC-3, such as AC field-induced switching and DC field-induced red tuning responses.

## 4. Conclusions

In this study, we report the phase behavior of BPLCs and electro-optic response of PSBPLCs depending on the concentration of the amorphous crosslinking agent PETA. The PETA in BPLC mixtures is mainly concentrated in the disclination lines, which inhibits the formation of the simple cubic BP-II structure and enhances the formation of the body-centered cubic BP-I structure. Only the isotropic to BP-I phase transition is observed for BPLC mixtures with 3–4 wt% amorphous crosslinker concentrations. The temperature window (ΔT) of BP-I widens from ~6.8 °C for BPLC-0 to ~15 °C for BPLC-4. The thermal stability of the BP-I structure in the polymer-stabilized BPLCs is greatly improved, especially in the low temperature range, and the BP-I phase is stable from 3 °C to 42 °C (ΔT > 39 °C), which is more than twice as stable as BPLC before photopolymerization (22 °C to 39 °C, ΔT = 16–17 °C). PSBPLC-3 and PSBPLC-4 show dynamic switching and red tuning responses of a selective reflection band when AC and DC voltages are applied, respectively. The phase behavior and dynamic response enable PSBLCs to be used in various photonic and display applications.

## Figures and Tables

**Table 1:** Composition of BPLC mixtures used in this study.

Sample	SL04151 (wt%)	PETA (wt%)	R1011 (wt%)	R811 (wt%)	E7 (wt%)
BPLC-0	9	0	4	21	66
BPLC-1	8	1	4	21	66
BPLC-2	7	2	4	21	66
BPLC-3	6	3	4	21	66
BPLC-4	5	4	4	21	66
BPLC-4-C6M	5	4 (C6M) ^1^	4	21	66

^1^ C6M monomer was used instead of PETA.

## Data Availability

Data are contained within the article.

## Supplementary Materials

The following are available online at https://www.mdpi.com/article/10.3390/nano12010048/s1, Figure S1: Transmission spectra and POM images of (a) BPLC-1 at (i) 47 °C (isotropic), (ii) 45.5 °C (BP-II), (iii) 43 °C (BP-I) and (iv) 22 °C (CLC phase) and (b) BPLC-2 at (i) 45 °C (isotropic), (ii) 42 °C (BP-II), (iii) 39 °C (BP-I) and (iv) 22 °C (CLC phase) in an aligned cell. Transmission spectra and POM images are monitored during cooling at a rate of 0.1 °C min^−1^. Scale bar in the POM images is 200 μm. 11.2 μm thick cells were used, Figure S2: (a) Reflective to transparent switching response of 4.7 μm thick PSBPLC-3 and (b) summary of peak positions while stepping up AC voltage to 110 V at 2 kHz, Figure S3. (a) AC field induced switching response between reflective and transparent states and (b) DC voltage induced redshift of the reflection band of 11.3 μm thick PSBPLC-4, Figure S4. Fractional residual lattice distortion Δλ_c_/λ_0_ as functions of electric field intensity E^2^ of PSBPLC-3 with various cell thicknesses (a) 4.7 µm, (b) 7.3 µm, and (c) 11.3 µm, Video S1: Switching response of PSBPLC-3 when an AC voltage (100 V at 2 kHz) is applied and removed, Video S2: Red tuning response of PSBPLC-3 when a DC voltage increases to 150 V.
