# Supplementary material for: Effect of Amorphous Crosslinker on Phase Behavior and Electro-Optic Response of Polymer-Stabilized Blue Phase Liquid Crystals
Source: Nanomaterials (Basel). 2021 Dec 24;12(1):48. doi: 10.3390/nano12010048 (PMC8746832; doi:10.3390/nano12010048)
Supplement: Supplementary file 1 [file nanomaterials-12-00048-s001.zip › nanomaterials-1514085 - Supplementary Materials v1/nanomaterials-1514085 - Supplementary Materials v1.pdf]

nanomaterials

Supplementary Materials

Effect of Amorphous Crosslinker on Phase Behavior and Electro-Optic Response of Polymer-Stabilized Blue Phase Liquid Crystals

Kyung Min Lee [1,2]*, Urice Tohgha [1,2], Timothy J. Bunning [1], Michael E. McConney [1] and Nicholas P. Godman [1,]*

[1] Materials and Manufacturing Directorate, Air Force Research Laboratory, Wright-Patterson AFB, OH 45433, USA; urice.tohgha.ctr@us.af.mil (U.T.); timothy.bunning@us.af.mil (T.J.B.); Michael.McConney.1@us.af.mil (M.E.M.)

[2] Azimuth Corporation, Fairborn, OH 45431, USA

* Correspondence: kyungmin.lee.3.ctr@us.af.mil (K.M.L.); nicholas.godman.2@us.af.mil (N.P.G.)

Supplementary Materials

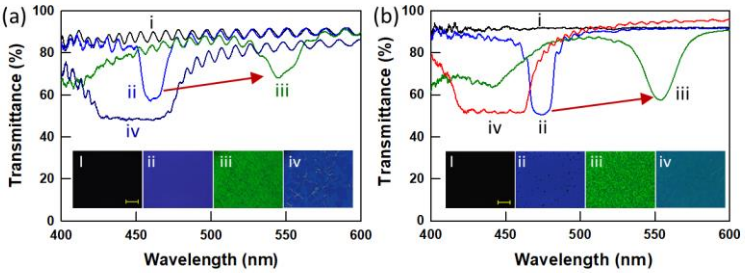

Figure S1. Transmission spectra and POM images of (**a**) BPLC-1 at (**i**) 47 °C (isotropic), (**ii**) 45.5 °C (BP-II), (**iii**) 43 °C (BP-I) and (**iv**) 22 °C (CLC phase) and (**b**) BPLC-2 at (**i**) 45 °C (isotropic), (**ii**) 42 °C (BP-II), (**iii**) 39 °C (BP-I) and (**iv**) 22 °C (CLC phase) in an aligned cell. Transmission spectra and POM images are monitored during cooling at a rate of 0.1 °C min^{-1}. Scale bar in the POM images is 200 μm. 11.2 μm thick cells were used.

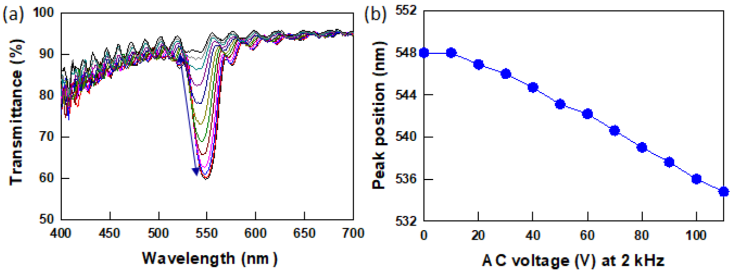

Figure S2. (**a**) Reflective to transparent switching response of 4.7 μm thick PSBPLC-3 and (**b**) summary of peak positions while stepping up AC voltage to 110 V at 2 kHz.

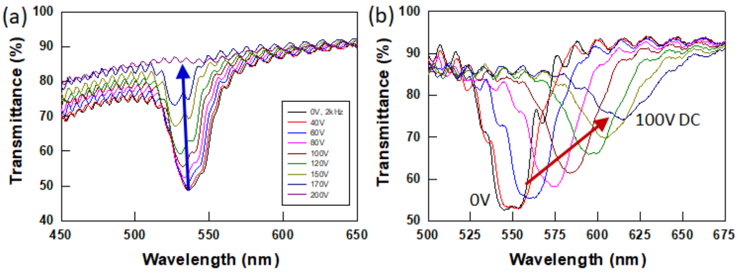

Figure S3. (**a**) AC field induced switching response between reflective and transparent states and (**b**) DC voltage induced redshift of the reflection band of 11.3 μm thick PSBPLC-4.

To obtain the electrostriction coefficient, the Kerr effect is neglected and only the residual lattice distortion along the field axis ($\Delta\lambda/\lambda_0 \sim RE^2$) is considered, where R is the electrostriction coefficient, $\Delta\lambda$ is the tuning range ($\Delta\lambda = \lambda_v - \lambda_0$), and E is the electric field strength [1]. PSBPLC-3 with various cell thicknesses exhibits electrical strain coefficients ranging from 1.2 to 1.7 × 10^{-3} μm^2 V^{-2} at low DC voltages and 3.6 to 8.4 × 10^{-4} μm^2 V^{-2} at high DC voltages, as can be seen in Figure S4. This slope change indicates the lattice distortion and requires further study.

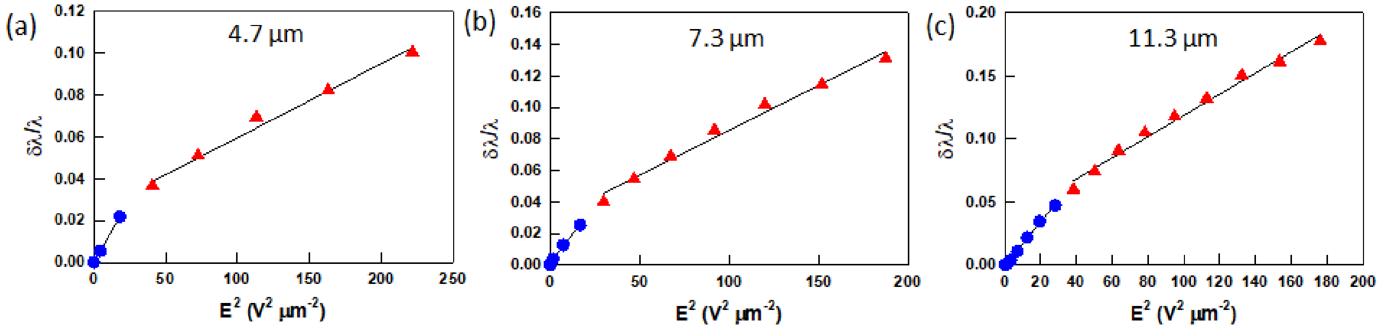

Figure S4. Fractional residual lattice distortion $\Delta\lambda_c/\lambda_0$ as functions of electric field intensity E^2 of PSBPLC-3 with various cell thicknesses (**a**) 4.7 μm, (**b**) 7.3 μm, and (**c**) 11.3 μm.

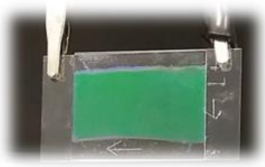

Video S1: Switching response of PSBPLC-3 when an AC voltage (100 V at 2 kHz) is applied and removed.

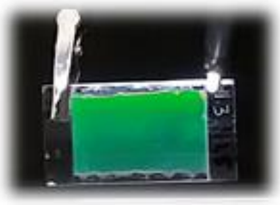

Video S2: Red tuning response of PSBPLC-3 when a DC voltage increases to 150 V.

References

1. Guo, D.-Y.; Chen, C.-W.; Li, C.-C.; Jau, H.-C.; Lin, K.-H.; Feng, T.-M.; Wang, C.-T.; Bunning, T. J.; Khoo, I. C.; Lin, T.-H. Reconfiguration of three-dimensional liquid crystalline photonic crystals by electrostriction. *Nat. Mater.* **2020**, *19*, 94–101
