# Supplementary material for: Effect of Amorphous Crosslinker on Phase Behavior and Electro-Optic Response of Polymer-Stabilized Blue Phase Liquid Crystals
Source: Nanomaterials (Basel). 2021 Dec 24;12(1):48. doi: 10.3390/nano12010048 (PMC8746832; doi:10.3390/nano12010048)
Supplement: Supplementary file 1 [file nanomaterials-12-00048-s001.zip › nanomaterials-1514085 - Supplementary Materials v1/nanomaterials-1514085 - Video Supplementary Materials.pptx]

## Slide 1
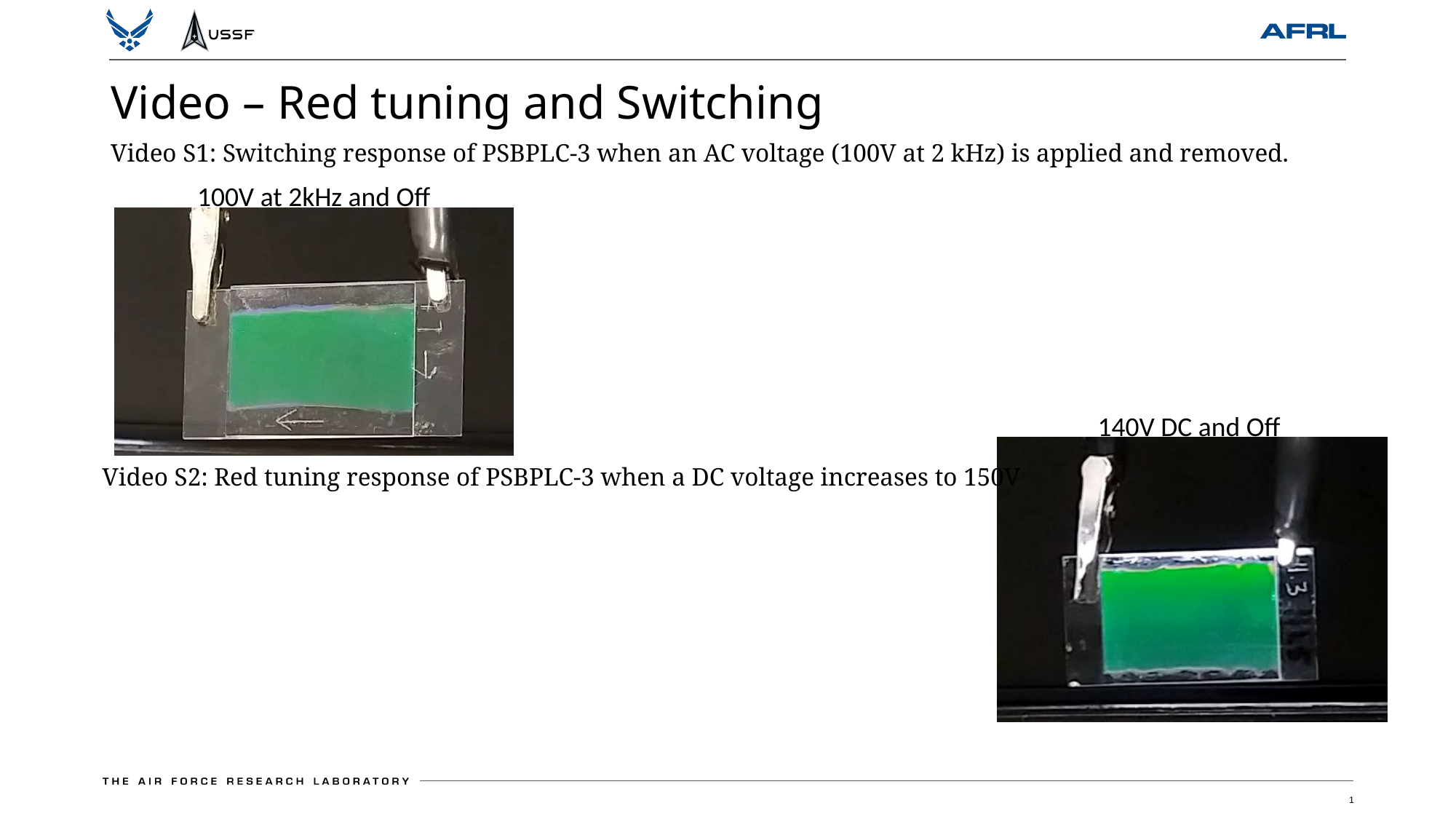

# Video – Red tuning and Switching
Video S1: Switching response of PSBPLC-3 when an AC voltage (100V at 2 kHz) is applied and removed.
100V at 2kHz and Off
140V DC and Off
Video S2: Red tuning response of PSBPLC-3 when a DC voltage increases to 150V
